# The more total cognitive load is reduced by cues, the better retention and transfer of multimedia learning: A meta-analysis and two meta-regression analyses

**DOI:** 10.1371/journal.pone.0183884

**Published:** 2017-08-30

**Authors:** Heping Xie, Fuxing Wang, Yanbin Hao, Jiaxue Chen, Jing An, Yuxin Wang, Huashan Liu

**Affiliations:** School of Psychology, Central China Normal University, Wuhan, China; Center for BrainHealth, University of Texas at Dallas, UNITED STATES

## Abstract

Cueing facilitates retention and transfer of multimedia learning. From the perspective of cognitive load theory (CLT), cueing has a positive effect on learning outcomes because of the reduction in total cognitive load and avoidance of cognitive overload. However, this has not been systematically evaluated. Moreover, what remains ambiguous is the direct relationship between the cue-related cognitive load and learning outcomes. A meta-analysis and two subsequent meta-regression analyses were conducted to explore these issues. Subjective total cognitive load (SCL) and scores on a retention test and transfer test were selected as dependent variables. Through a systematic literature search, 32 eligible articles encompassing 3,597 participants were included in the SCL-related meta-analysis. Among them, 25 articles containing 2,910 participants were included in the retention-related meta-analysis and the following retention-related meta-regression, while there were 29 articles containing 3,204 participants included in the transfer-related meta-analysis and the transfer-related meta-regression. The meta-analysis revealed a statistically significant cueing effect on subjective ratings of cognitive load (*d* = −0.11, 95% CI = [−0.19, −0.02], *p* < 0.05), retention performance (*d* = 0.27, 95% CI = [0.08, 0.46], *p* < 0.01), and transfer performance (*d* = 0.34, 95% CI = [0.12, 0.56], *p* < 0.01). The subsequent meta-regression analyses showed that *d*_SCL_ for cueing significantly predicted *d*_retention_ for cueing (β = −0.70, 95% CI = [−1.02, −0.38], *p* < 0.001), as well as *d*_transfer_ for cueing (β = −0.60, 95% CI = [−0.92, −0.28], *p* < 0.001). Thus in line with CLT, adding cues in multimedia materials can indeed reduce SCL and promote learning outcomes, and the more SCL is reduced by cues, the better retention and transfer of multimedia learning.

## Introduction

As one of the material-oriented interventions in multimedia learning, cueing affects learners’ cognitive processing and learning outcomes [1–3]. The term cueing refers to the non-content information (e.g., arrows, color coding, highlighting) added in learning materials to attract learners’ attention and to further promote their selection, organization, and integration of instructional elements [4, 5]. A large body of research has shown that adding cues in multimedia materials facilitated retention and transfer of learning [4, 6–10]. Two recent meta-analyses have also confirmed the robustness of the cueing effect on learning outcomes [11, 12]. Many researchers have given an explanation from the perspective of cognitive load theory (CLT) [13], maintaining that cues could reduce learners’ overall cognitive load and help avoid overload, thus contributing to their learning performance. Nevertheless, two crucial problems that remain to be solved are (a) whether the cues really reduce total cognitive load, and (b) how a reduction in cueing-related cognitive load may be related to learning outcomes. In the current study, a meta-analysis and two following meta-regression analyses focused on these issues.

### The measurement of cognitive load

Cognitive load is generally defined as a multidimensional construct representing the cognitive demands associated with performing a specific task [14, 15]. In the 1980s Sweller [16] proposed cognitive load theory, which articulated the association between cognitive resources and task demands in creating cognitive load. Learners will consume their cognitive resources as long as they are performing a task, leading to cognitive load. In this model, working memory is a cognitive resource, but is a limited one; only a small fraction of elements can be consciously handled per unit time, especially when they are novel or unfamiliar. However, long-term memory provides the ability to circumvent the limitation of working memory with the help of schemas. Schemas are cognitive constructs in which multiple elements are incorporated into a single element, which can then be processed automatically. Consequently, the construction and automation of schemas are the main goals of instruction.

In the field of educational research, cognitive load theory [13, 17] is mainly used to explain the effects of various forms of instructional design. According to this theory, intrinsic cognitive load (ICL) is not directly affected by instructional design. It is related to element interactivity in learning materials and learners’ prior knowledge. Element interactivity has been regarded as the primary, representative mechanism of ICL for quite a long time. The level of ICL of a specific task is usually assumed to depend on the level of element interactivity. An element can be anything that will be or has been presented, for example a concept or a procedure. Instructional materials with low element interactivity allow single (or several) element(s) to be processed with little or even no reference to other elements, thus resulting in a low ICL; however, high element interactivity materials contain elements that heavily interact with each other and cannot be processed separately, leading to a high ICL. Extraneous cognitive load (ECL) is concerned with the quality of instructional design. It is detrimental to the processes of schema construction and automation and thus will hinder learning. Germane cognitive load (GCL) is directly beneficial to learning. It can be imposed by the cognitive processes of active schema construction such as classifying, inferring, and organizing. The total cognitive load during information processing is the sum of the three kinds of cognitive loads. One important objective of instructional design is to ensure that the total cognitive load is within the learner’s cognitive capacity, in order to avoid cognitive overload [14].

Different techniques have been used to measure cognitive load, mainly including subjective rating scales, dual-task performance, and physiological measures [14, 18–20]. Pass [21] introduced the mental effort scale, which was a modified version of Bratfisch, Borg, and Dornic’s scale [22] for measuring perceived task difficulty. Pass’s 9-point mental effort scale included one item that asked learners to report how much mental effort they invested when learning the material. Since then, the mental effort or perceived difficulty scale has been widely used in research in the field of learning and instruction because it is easy to administer, is non-invasive, and has good reliability and validity [14].

Bratfisch, Borg and Domic’s [22] mental effort or perceived difficulty scale for measuring subjective cognitive load (SCL) has been modified in several ways in previous studies. This heterogeneity reflects differences in terms of scale items, number of scale units, and the timing of measurement [19]. Concerning items, Pass [21] asked participants to report their invested effort on a 9-point Likert scale by translating the perceived amount of mental effort into a numerical value. Other researchers have required learners to estimate how difficult (or easy) it was for them to learn from the instruction [8, 10, 23]. Some studies combined mental effort with perceived difficulty to measure cognitive load [24–27], whereas others added items measuring something that was different than cognitive load (e.g., “It was important to me to do well at this task” [28]). As for number of units, these scales have ranged from 9 points to 5 points (See Table 1). In most studies the questionnaire was given to the learners after learning had taken place [4, 8, 25], whereas other studies presented it during the learning process [29, 30].

**Table 1:** Studies included in the meta-analysis and meta-regression analyses.

Study	Year	Sample size	Sample characteristic	Type of cueing	Instructional domain	Number of SCL-scale units	Timing of SCL measurement	SCL-scale items	Data source for SCL
Kalyuga, Chandler, & Sweller [8]	1999	*N* = 16	first-year apprentices and trainees	color coding	physics	7-point	after learning	One item: Estimate how easy or difficult the instructions were to understand.	difficulty
Jamet, Gavota, & Quaireau [25]	2008	*N* = 102	college students	color change or step-by-step presentation of diagram elements	biology	a continuous scale with a maximum of 100	after learning	Two items: (a) mental effort (‘‘Little effort needed to learn the document”) and (b) ease of learning (‘‘This presentation helps me to memorize information” and ‘‘This presentation helps me to focus on the relevant information”).	combination of mental effort and task difficulty
De Koning, Tabbers, Rikers, & Pass [38]	2010	*N* = 37	college students	spotlight	biology	9-point	after learning	One item: How much mental effort they had invested in studying the animation.	mental effort
Berthold & Renkl [24]	2009	*n* = 85	high school students	color coding and flashing	math	9-point	after learning	Five items: (a) How easy or difficult do you consider probability theory at this moment? (b) How easy or difficult is it for you to work with the learning environment? (c) How easy or difficult is it for you to distinguish important and unimportant information in the learning environment? (d) How easy or difficult is it for you to collect all the information that you need in the learning environment? (e) Indicate on the scale the amount of effort you invested to understand the last example task.	combination of mental effort and task difficulty
Moreno, Reisslein, & Ozogul [27]	2010	*N* = 159	Middle school students	APA signaling, arrow signaling	physics	5-point	after learning	One item: Asking participants to rate their difficulty and mental effort perceptions on a 5-point scale [21].	combination of mental effort and task difficulty
Johnson, Ozogul, & Reisslein [44]	2015	*N* = 250	Middle school students	APA signaling, arrow signaling	physics	5-point	after learning	Six items: Evaluations of the difficulty of the program (such as ‘It was difficult to learn from this program’ and ‘The topics that were covered in the lesson were difficult)	difficulty
Johnson, Ozogul, Moreno, & Reisslein [54]	2013	*n* = 196	Middle school students	APA signaling, arrow signaling	physics	5-point	after learning	Two items: (a) The lesson was difficult. (b)Learning the material in the lesson required a lot of effort.	combination of mental effort and task difficulty
Arslan-Ari [45]	2013	*N* = 200	college students	Label, picture, label and picture	biology	9-point	after learning	One item: Participants were asked to rate how much mental effort they invested in studying the material.	mental effort
De Koning, Tabbers, Rikers, & Paas [39]	2010	*N* = 73	college students	spotlight	biology	9-point	after learning	One item: Participants were asked to rate how much effort it took them to complete a task.	mental effort
Huk, Steinke, & Floto [23]	2010	*N* = 247	college students	either color coding of special parts or a written presentation of technical terms	biology	5-point	after learning	Two items: (a) The computer animation ‘‘ATP-Synthase” is easy to comprehend. (b) The computer animation ‘‘ATP-Synthase” is easy to survey.	difficulty
Spanjers, Van Gog, Wouters, & Van Merriënboer [55]	2012	*n* = 78	high school students and pre-university students	pausing and temporal cueing	math	9-point	after learning	One item: Students were asked to rate how much mental effort they invested in studying each animation	mental effort
Zhao [56]	2013	*n* = 96	postgraduate students	color, bold	physics	7-point	after learning	One item: Mental effort measure developed by Paas [21].	mental effort
De Koning, Tabbers, Rikers, & Paas [4]	2007	*N* = 40	college students	spotlight	biology	9-point	after learning	One item: Mental effort measure developed by Paas [21].	mental effort
Jamet & Fernandez [34]	2016	*N* = 51	college students	green arrows	computer science	a continuous scale with a maximum of 20.	after learning	One item: Learning with the tutorial took a great deal of mental effort.	mental effort
Ouwehand, Van Gog, & Paas [30]	2015	*n* = 92	children	gesture cues, symbolic cues	physics	9-point	during learning	One item: Mental effort scale adapted from Paas [21].	mental effort
Moreno [26]	2007	*n* = 235	pre-service teachers	instructional videos and animations	social science	5-point	after learning	Two items: (a) How difficult was it to learn about essential teaching skills with the computer program? (b) How much effort did you have to invest to learn about essential teaching skills with the computer program?	combination of mental effort and task difficulty
Paik & Schraw [46]	2013	*N* = 65	college students	representational animation and directive animation (color, arrows)	physics	n/a	after learning	Two items: (a) How difficult was it to learn about the flushing toilet tank from the presentation? (b) How much mental effort was required to learn about the flushing toilet tank from the presentation?	combination of mental effort and task difficulty
Wang, Duan, Zhou, & Chen [37]	2015	*N* = 51	college students	color	physics	9-point	after learning	One item: The mental effort measure developed by Paas [21].	mental effort
Zou [57]	2013	*n* = 180	college students and postgraduate students	color, arrow	physics	9-point	after learning	One item: Mental effort measure developed by Paas [21].	mental effort
Yung & Paas [32]	2015	*N* = 133	seventh grade students	APA	biology	5-point	after learning	One item: Measurement of cognitive load consisted of two types of subjective measures: perceived task difficulty and perceived amount of invested mental effort [21].	combination of mental effort and task difficulty
De Koning, Tabbers, Rikers, & Paas [40]	2011	*N* = 84	college students	spotlight	biology	9-point	after learning	One item: The perceived amount of mental effort invested in studying the animation was indicated on the 9-point rating scale developed by Paas [21].	mental effort
Ozcelik, Karakus, Kursun, & Cagiltay [10]	2009	*N* = 48	college students	color coding	biology	7-point	after learning	One item: Participants were requested to rate how easy or difficult it was for them to understand the instructions.	difficulty
Tabbers, Martens, & Van Merriënboer [58]	2000	*N* = 151	college students	color coding	educational psychology	9-point	during learning	One item: Mental effort scale developed by Paas [21].	mental effort
Song & Bruning [35]	2015	*N* = 147	college students	titles, headings, previews, summary statements, logical connectives and typographical cues	geography	5-point	after learning	One item: How much effort did you put into learning the information?	mental effort
Crooks, Cheon, Inan, Ari, & Flores [36]	2012	*N* = 135	college students	color change, animated pointer	biology	5-point	after learning	One item: Please indicate how much mental effort you invested in this computer lesson.	mental effort
Van Gog, Jarodzka, Scheiter, Gerjets, & Paas [59]	2009	*n* = 66	college students	model’s eye movements in examples	problem-solving task	9-point	after learning	One item: The perceived amount of mental effort invested in observing the examples and solving the test problems was indicated on the scale developed by Paas [21].	mental effort
Jarodzka, Van Gog, Dorr, Scheiter, & Gerjets [60]	2013	*N* = 75	college students	dot display, spotlight display	biology	9-point	after learning	One item: How much effort did you invest to complete this task?	mental effort
Seufert & Brünken [61]	2006	*N* = 88	college students	hyperlinking, explaining the relations of corresponding structures more or less explicitly	biology	7-point	after learning	One item: Cognitive load was measured by a subjective 7-point rating scale that ranges from very low to very high mental effort (adapted from Paas [21]).	mental effort
Tabbers, Martens, & Van Merriënboer [29]	2004	*N* = 111	college students	color coding	educational psychology	9-point	during learning	One item: Mental effort measure developed by Paas [21].	mental effort
Zhou [62]	2014	*n* = 180	college students	color, arrow	physics	7-point	after learning	One item: Mental effort measure developed by Paas [21].	mental effort
Amadieu, Mariné, & Laimay [63]	2011	*N* = 36	college students	zoom	biology	9-point	after learning	One item: Please indicate how much mental effort you invested to learn the mechanism of the Long Term Potentiation.	mental effort
De Koning, Tabbers, Rikers, & Paas [41]	2011	*N* = 90	high school students	spotlight	biology	9-point	after learning	One item: Mental effort measure developed by Paas [21].	mental effort

*Note*. Studies ranked according to pooled Cohen’s *d* magnitude on SCL.

*n* = number of partial participants in an article. *N* = number of whole participants in an article. SCL = subjective cognitive load.

After learning = SCL invested in the learning phase was measured after learning had taken place.

During learning = SCL invested in the learning phase was measured several times during the learning process.

n/a = not available. APA = animated pedagogical agent.

What kind of cognitive load does mental effort or perceived difficulty (SCL) represent: ICL, ECL, GCL, or total cognitive load? We assert that mental effort or perceived difficulty is more likely to represent the total cognitive load for the following reasons: (1) Mental effort is defined as “the aspect of cognitive load that refers to the cognitive capacity that is actually allocated to accommodate the demands imposed by the task” [14]. It is influenced by three factors: task environment characteristics, learner characteristics, and their interaction. The combination of these factors is more likely to represent the total cognitive load, rather than a specific cognitive load. (2) A mental effort or perceived difficulty scale does not specify which aspect of the learning experience the participants are required to rate. Rather, participants report their overall feelings about learning. (3) This position is consistent with that of Pass and colleagues [14, 31, 32] and other researchers [33–35] who have considered all measures of mental effort or perceived difficulty to assess overall cognitive load, rather than its constituent components (i.e., intrinsic, extraneous, germane).

### Effects of cueing on cognitive load and multimedia learning performance

Multimedia learning materials usually have high element interactivity, and learners often have no idea how to quickly search and process the correct elements in the limited time. In this context, the total cognitive load that learners bear can easily exceed the limited capacity of cognitive resources. In order to avoid cognitive overload during the learning process, many researchers add some non-content information (i.e., cues) in the learning material to guide the learners’ attention and reduce their total cognitive load. For example, Jeung, Chandler, and Sweller [7] studied participants who were learning geometry. In the experimental group, when the participants heard any comment about a certain rectangle (e.g., “area of the rectangle MADE”), this rectangle (e.g., MADE) in the picture would flash. De Koning et al. [4] added visual cues in the animation presented to the experimental group. When explaining a certain element in the cardiovascular system, all elements in the animation except this certain element would be darkened, which could be thought of as a spotlight-effect. Analogously, when experimental group participants in Lin and Atkinson’s [28] study were learning information about the rock cycle, red arrows would appear and point to the element being discussed. However, no such cues were presented to the control groups in these studies.

According to CLT, cues should prevent cognitive overload [4]. However, this has not yet received consistent support from empirical research. Some studies have found that cues could reduce students’ perceived amount of invested mental effort or perceived task difficulty. For instance, Kalyuga et al. (Experiment 2) [8] found that using color coding reduced searches for diagrammatic referents in the text and ameliorated split-attention effects, resulting in lower perceived difficulty. Berthold and Renkl [24] showed that participants who were provided color coding and flashing reported significantly less SCL than their counterparts without such aids. However, a larger number of studies have found that cues do not produce a significant reduction in SCL [29, 36, 37]. Ozcelik et al. [10] found there was no significant difference in the perceived difficulty of the instruction (chemical synapses) between the color-coded group and the conventional group. A series of studies by De Koning and colleagues also did not find any effect of cues on mental effort [4, 38–41]. In sum, there is a lack of cogent evidence about whether cues affect cognitive load.

Compared to research on cues and SCL, results concerning the impact of cues on learning outcomes can be considered to be a bit more consistent; that is, adding cues in multimedia materials can improve learning performance. Ozcelik, Karakus, Kursun and Cagiltay [10] discovered that color coding facilitated learners’ memory and comprehension of chemical synapses, revealing higher scores on retention and transfer tests, though the cues did not influence perceived difficulty. By now, a positive cueing effect on learning outcomes has been shown in numerous studies in various instructional domains, such as math [7], biology [4, 10, 39], engineering [42, 43], psychology [6], and physics [8, 44]. Though some studies did not find an improvement in learning outcomes due to cues [36, 38, 45], the authors of two recently published meta-analyses concluded that overall, cues indeed improved learning performance [11, 12].

### Research questions and Hypotheses

The first question is whether cueing can indeed lower learners’ total cognitive load and help avoid cognitive overload, as cognitive load theory predicts. The present study used meta-analysis to assess whether cueing reduces cognitive load, as measured by SCL (mental effort, perceived difficulty, or their combination) and improves learning (retention and transfer). In addition, the present study calculated pooled effect sizes for the impact of cues on a retention test and transfer test in order to compare the results with those of two related meta-analyses [11, 12] that adopted different inclusion criteria from ours. Our first research question about effects of cueing on total cognitive load was not considered in the two recently published meta-analyses, which only looked at the effects of cueing on learning outcomes.

Supposing the findings of the meta-analysis do suggest that cueing reduces total cognitive load, then the following question is about the relationship between cueing-related cognitive load and learning outcomes. One of the solutions is to use correlation or regression analysis to explore this question. For example, a few studies have found a significant negative correlation between SCL and learning performance [23, 46]. Nevertheless, the vast majority of empirical researchers have separately analyzed the effects of cueing on cognitive load or on learning outcomes [4, 25, 29, 39], leading to a lack of evidence needed to make direct inferences about their relations. An alternative solution is to use meta-regression to examine the predictive effect of cueing-related cognitive load on learners’ performance. If total cognitive load is reduced by cues, there is every reason to predict better learning outcomes (e.g., better retention or transfer performance) according to CLT. Two meta-regression analyses were used to test this assumption. This statistical method synthesizes correlational results across studies. In this case the correlational data that were included were those describing the relation between cueing-related SCL and scores on retention or transfer tests, respectively.

According to CLT, we expected that (a) cues can reduce SCL (Hypothesis 1) and (b) promote scores on retention (Hypothesis 2a) and transfer (Hypothesis 2b) in multimedia environments, and further, (c) the more SCL is reduced by cues, the better retention (Hypothesis 3a) and transfer (Hypothesis 3b) of multimedia learning. Hypotheses 1, 2a and 2b were tested using meta-analysis; Hypotheses 3a and 3b were tested using meta-regression analyses.

## Methods

The PRISMA (Preferred Reporting Items for Systematic Reviews and Meta-Analyses) guidelines were followed (S1 Checklist).

### Literature search

To identify relevant studies on the effects of cueing in multimedia learning, a systematic literature search was conducted by searching the electronic databases PsycINFO, Education Research Complete, Science Direct, PubMed, ProQuest, and China National Knowledge Infrastructure (CNKI). Search engines such as Google Scholar and the reference lists of the identified studies were also used. The search keywords were “cue,” “cueing,” “signaling,” and “color coding” with different combinations of “multimedia learning,” “cognitive load,” “mental effort,” “retention,” and “transfer.” The search was limited to the period between January 1995 and March 2016. To minimize the file drawer problem, we (1) tried to contact some researchers to provide the details of their unpublished studies (e.g., dissertations or conference papers) through email, and also (2) assessed publication bias statistically (see below).

### Study selection

Journal articles as well as dissertations and conference presentations would be selected. The following inclusion criteria for the retrieved articles were adopted for the meta-analysis. The studies were included if (a) they were based on an experimental design; (b) multimedia learning materials were used, that is, the materials simultaneously contained words (e.g., on-screen text, narration) and pictures (e.g., diagrams, videos, animations); (c) both an experimental group with cues and a control group without cues were compared; (d) sufficient quantitative data (e.g., means, standard deviations and *n*; *t* test or *F* test values) were reported to calculate effect size; and (e) the effect size did not go beyond three *SD* of the mean of all effect sizes to alleviate the effect of outliers representing extreme values [47], which would make mean values unrepresentative of the literature as a whole. Studies were excluded if they did not meet the inclusion criteria mentioned above.

It was important to emphasize that different studies might adopt multiple dependent variables to measure (total) cognitive load. A basic assumption of meta-analysis is the independence of effects, and the inclusion of multiple dependent variables in each study would not conform to this assumption [48]. Thus to abide by this assumption and avoid the potential deviation due to dependencies between effect sizes introduced by multiple variates per study, the following criteria were also used:

1. The study measured SCL invested in the learning phase rather than in the test phase, regardless of whether the scale was presented after learning had taken place or during the learning process.
2. We chose data representing mental effort during the learning process as long as they were provided independently, regardless of whether or not other indexes of cognitive load were available.
3. If no data representing mental effort were included but data on the perceived difficulty of the instruction were provided independently, this result was included.
4. If the only available data represented the combination of mental effort and perceived difficulty, rather than independent data for each, the combined result was included.
5. Except for the three situations above, studies using any other measuring methods were disregarded. For example, Lin and Atkinson’s [28] study, which used a subscale named Effort to assess mental effort as well as other constructs (e.g., It was important to me to do well at this task.), was excluded from our study.

### Data extraction

Data with respect to SCL ratings for both experimental and control groups were extracted by two of the authors (HX, YH) and checked by another (JC). Discrepancies were resolved through discussion.

### Statistical analyses

As for analyses of retention and transfer performance, the included studies reported both SCL and a learning outcome (i.e., SCL and retention test, or SCL and transfer test, or SCL and both). Because the data included in this study were continuous data with no consistent unit, we chose Cohen’s *d* as the standardized estimate of effect size for each article [49]. Specifically, Cohen’s *d* was calculated as the mean score difference in SCL ratings or learning outcomes between cueing (experimental) and no-cueing (control) groups. When the studies reported multiple experiments or multiple conditions, the data were merged to compute one pooled effect size in order to minimize the deviation of results caused by a large number of effect sizes and disproportionate weight if not pooled [50]. For instance, Jamet et al. [25] conducted a 2 (Salience: salient, non-salient) × 2 (Display: sequential, static) between-subjects design, which could have generated two effect sizes (one effect size per condition of Display), but we combined these two into a pooled study-level effect size through CMA (see below). Study-level effect sizes were then averaged to obtain an overall average effect size. The 95% confidence interval (95% CI) of each effect size was also calculated. For Cohen’s *d*, the direction of the effect size was negative if the SCL rating or learning performance of the cueing group was lower than that of the no-cueing group. An effect size of ±0.2 was considered to be small, ±0.5 moderate, and ±0.8 large [51]. A random-effects model was preliminarily selected to calculate the pooled effect sizes and their 95% CIs because articles included in the present study differed in a number of variables (e.g., groups of participants, research methods), potentially resulting in a heterogeneity of results among studies.

Data were analyzed using the Comprehensive Meta-Analysis (CMA) 2.0 software (https://www.meta-analysis.com/). We calculated the *Q* statistic with its *p* value to test whether the random-effects model used in this study was reasonable. A statistically significant *Q* value indicates that it would be better to calculate effect sizes based on the random-effects model [48, 52]. Regarding publication bias, Egger’s linear regression test [53] was used. Through this test, a regression equation can be created with the standard normal deviate of each study as the dependent variable and the estimate’s precision of each study as the independent variable. The intercept of the regression equation provides a measure of publication bias. The smaller its deviation from zero the less pronounced the bias.

## Results

### Included studies

**Fig 1:**
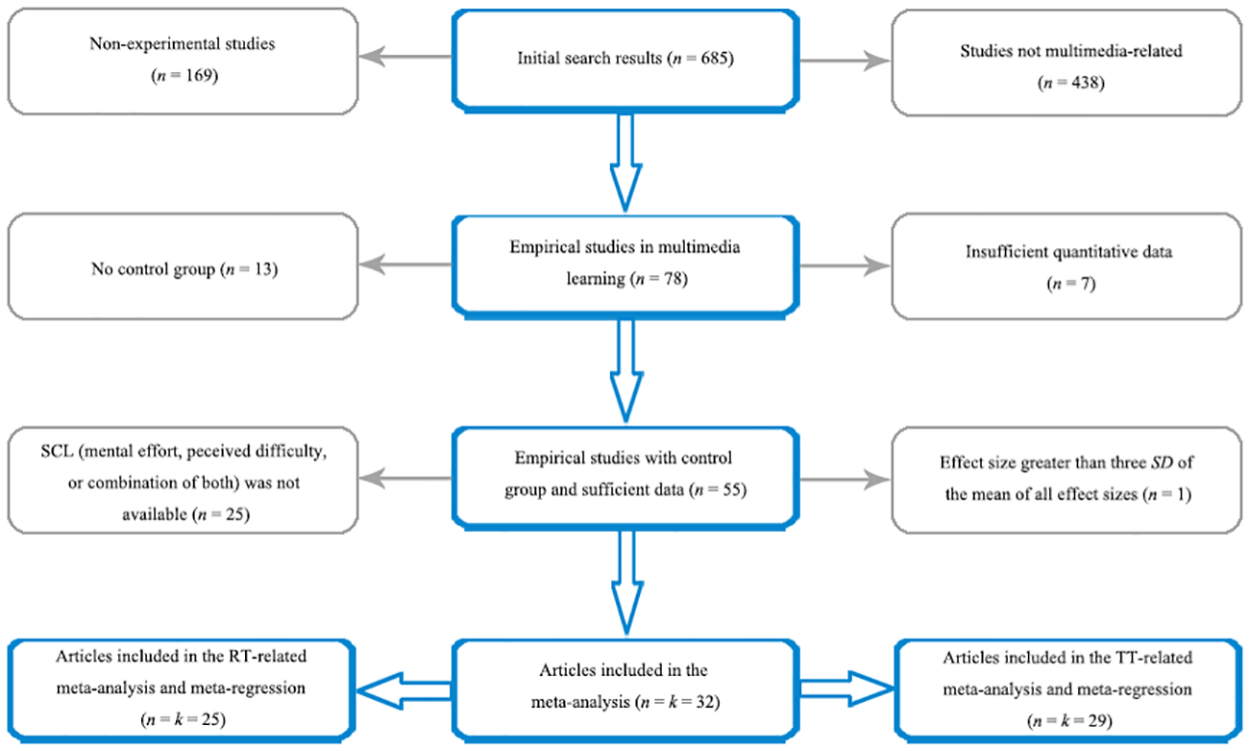
Flow chart of initial literature search and study selection process. *Note*. *n* = number of articles, *k* = number of effect sizes, RT = retention test, TT = transfer test.

Results of the initial literature search and study selection are shown in Fig 1. A total of 32 articles that met the inclusion criteria were finally included and analyzed. There were 27 articles obtained from journals, 4 from dissertations, and 1 from an academic conference. Twenty-eight articles were written in English, and 4 in Chinese. Accordingly, 32 study-level effect sizes with respect to SCL were computed, involving 3,597 participants (See Table 1 and S1 Dataset). Twenty-five study-level effect sizes regarding retention containing 2,910 participants and 29 study-level effect sizes regarding transfer encompassing 3,204 participants were also computed.

### Effects of cueing on SCL and learning outcomes

**Fig 2:**
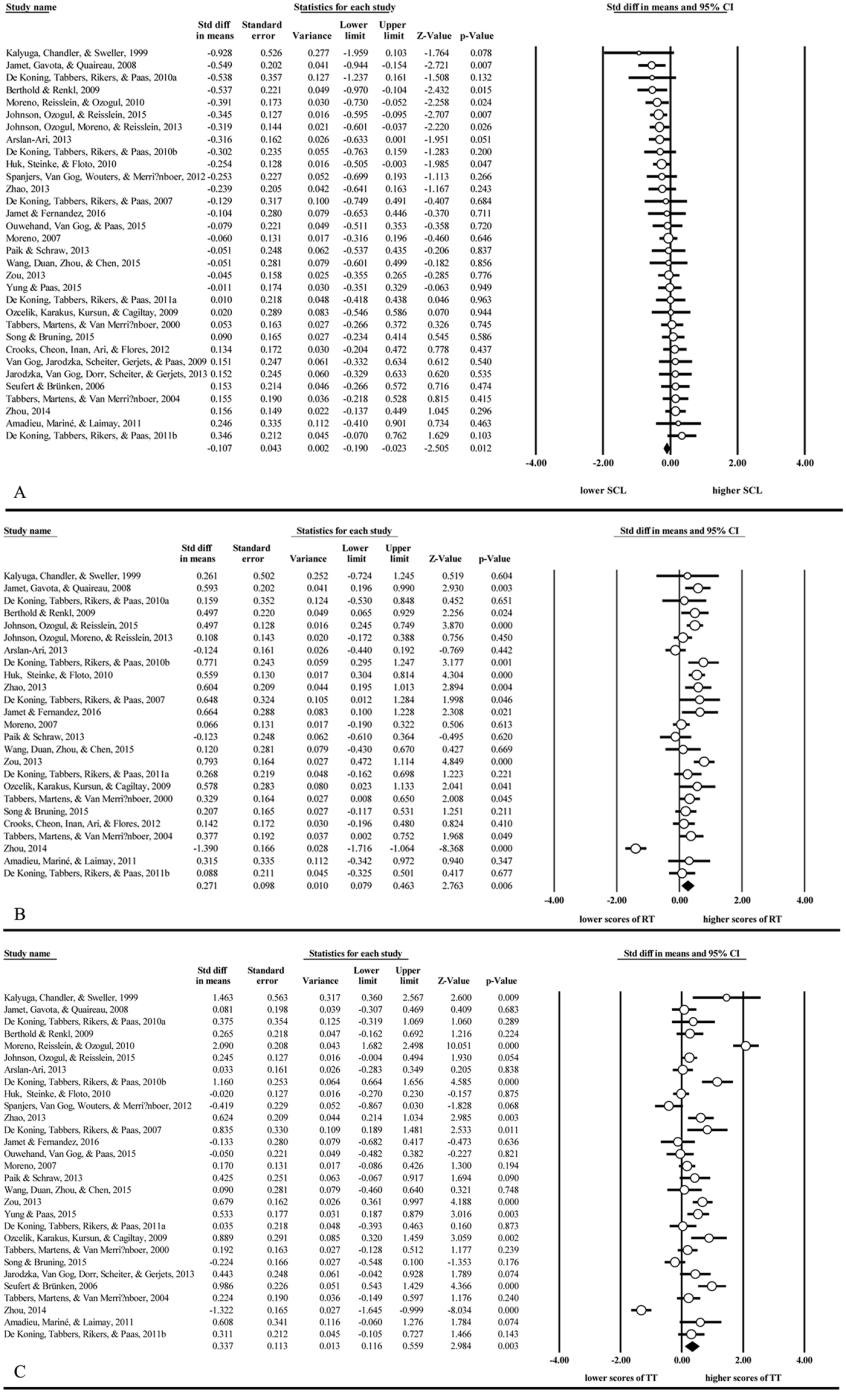
Forest plots of the meta-analysis concerning each index. *Note*. **(A)** Meta-analysis of SCL. **(B)** Meta-analysis of retention test. **(C)** Meta-analysis of transfer test. *Note*. RT = retention test, TT = transfer test.

Table 2 presents the results of cueing on SCL as well as learning outcomes, and forest plots of the meta-analysis with respect to each index are shown in Fig 2. Concerning SCL, the meta-analysis based on the random-effects model revealed that the overall pooled effect size was small but statistically significant (*d* = −0.11, 95% CI = [−0.19, −0.02], *p* < 0.05). Thus is in line with CLT, adding cues in multimedia materials reduced learners’ perceived total cognitive load.

**Table 2:** Main effects of cueing on SCL and learning outcomes based on the random-effects model.

	*k*	*N*	Cohen’s *d*	95% CI	*Q* statistic		Egger’s test	
					value	*p*	intercept	*p*
SCL	32	3,597	−0.11*	[−0.19, −0.02]	45.20	< 0.05	0.15	> 0.05
Retention	25	2,910	0.27**	[0.08, 0.46]	147.02	< 0.001	0.83	> 0.05
Transfer	29	3,204	0.34**	[0.12, 0.56]	250.10	< 0.001	3.11	> 0.05

*Note*. The *p* value of Egger’s test is two-tailed.

*k* = number of effect sizes, *N* = total number of participants, CI = confidence interval.

**p < 0.01,

*p < 0.05.

Concerning learning outcomes, there were small-to-medium cueing effects for both retention (*d* = 0.27, 95% CI = [0.08, 0.46], *p* < 0.01) and transfer (*d* = 0.34, 95% CI = [0.12, 0.56], *p* < 0.01). These results are consistent with the previous two meta-analyses; adding cues in multimedia materials facilitated retention and transfer of learning.

### Potential relationships between cueing-related SCL and multimedia learning outcomes

If cues reduce SCL, would this lead to a better retention or transfer of learning as CLT would expect? To clarify this question, we conducted two parallel meta-regression analyses to investigate the potential relationships between SCL and scores for retention or transfer. In both analyses SCL (*d*_SCL_) effect sizes were used as the predictor, and retention test (*d*_retention_) and transfer test (*d*_transfer_) effect sizes as dependent variables (Table 3; S2 Dataset). The data sources were the same as those used for retention- and transfer-related meta-analysis.

**Table 3:** Main findings of the studies included in the retention-related and transfer-related meta-regression analyses.

Predictor→Dependent Variables	*k*	*N*	β	*SE*_β_	95% CI	*Z*
*d*_SCL_→*d*_retention_	25	2,910	−0.70***	0.16	[−1.02, −0.38]	−4.30
*d*_SCL_→*d*_transfer_	29	3,204	−0.60***	0.16	[−0.92, −0.28]	−3.70

*Note*. *k* = number of effect sizes, *N* = total number of participants.

β = point estimate of slope, *SE*_β_ = standard error of β, CI = confidence interval.

***p < 0.001.

**Fig 3:**
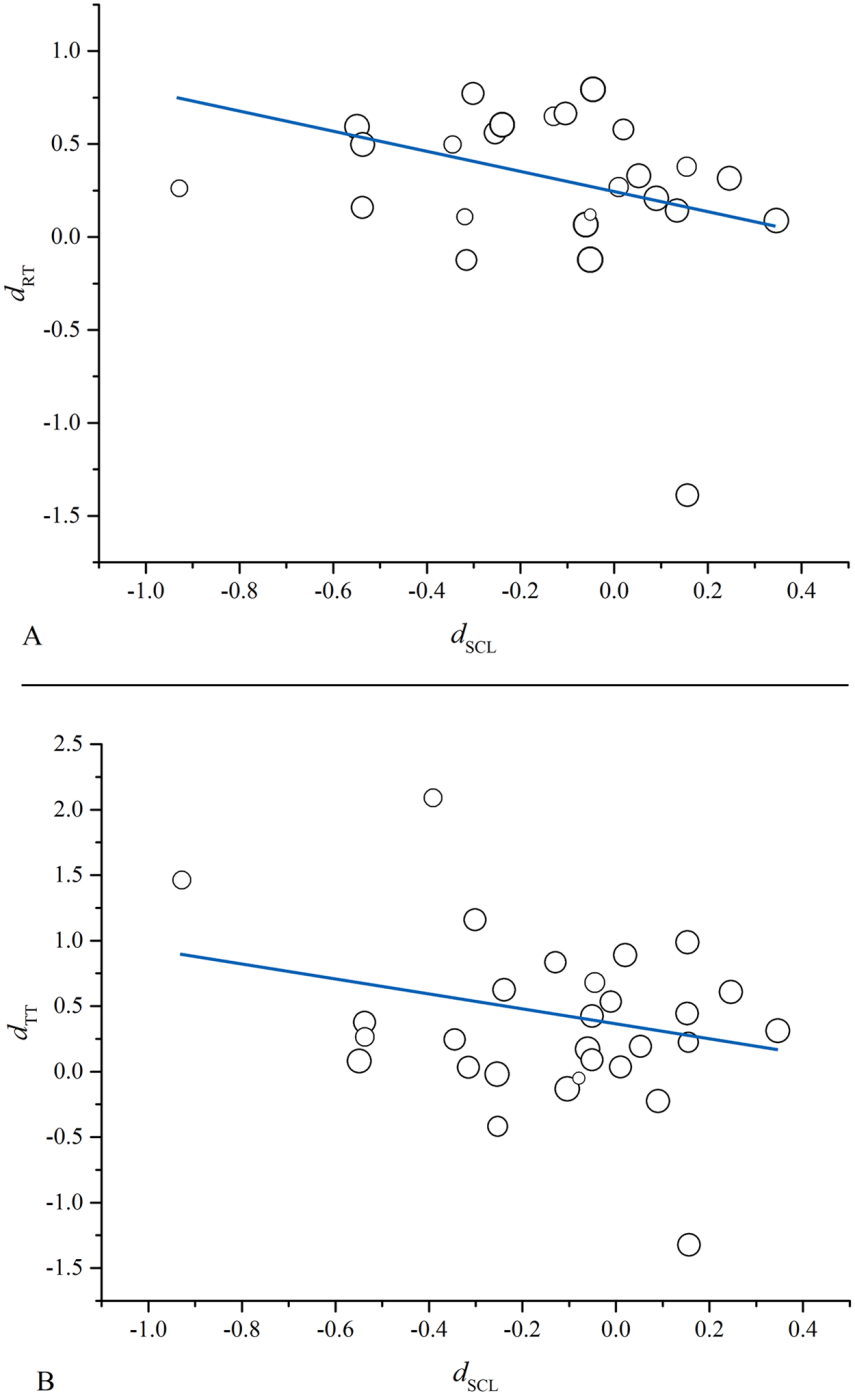
Retention-related and transfer-related meta-regression analyses. ***Note*. (A)** Regression of *d*_SCL_ on *d*_retention_. **(B)** Regression of *d*_SCL_ on *d*_transfer_. *Note*. RT = retention test, TT = transfer test. The size of the circle is proportional to study weight.

First, a meta-regression with *d*_SCL_ as the predictor and *d*_retention_ as the dependent variable was conducted (Table 3; Fig 3A). The result revealed that the effect of cueing represented by *d*_SCL_ could significantly predict the effect of cueing represented by *d*_retention_ (β = −0.70, 95% CI = [−1.02, −0.38], *p* < 0.001). Thus in line with CLT, the more SCL was reduced by cues, the better retention of learning. Similarly, a meta-regression with *d*_SCL_ as the predictor and *d*_transfer_ as the dependent variable was conducted (Table 3; Fig 3B). The result showed that *d*_SCL_ significantly predicted *d*_transfer_ (β = −0.60, 95% CI = [−0.92, −0.28], *p* < 0.001). Thus also in line with CLT, the more SCL was reduced by cues, the better transfer of learning.

### Publication bias analyses

As shown in Table 2, the *p* values of the *Q* statistic were significant (*Q*_SCL_ = 45.20, *p* < 0.05; *Q*_retention_ = 147.02, *p* < 0.001; *Q*_transfer_ = 250.10, *p* < 0.001), indicating significant heterogeneity in the results. Thus, using a random-effects model in this study was appropriate. Egger’s linear regression test was not significant, suggesting that publication bias was an unlikely influence on the results.

## Discussion

A meta-analysis and two subsequent meta-regression analyses were conducted in the present study to test whether the addition of cues can reduce learners’ subjective cognitive load and promote learning outcomes in a multimedia environment, and further, to explore the relationship between cognitive load and learning performance in this learning context. Consistent with Hypothesis 1, learners in the cueing condition reported a lower perception of cognitive load than learners in the no-cueing condition (*d* = −0.11, *p* < 0.05), revealing that cues could reduce subjective cognitive load. Results from retention-related meta-analysis and transfer-related meta-analysis also showed that adding cues in multimedia materials facilitated retention and transfer of learning significantly, thus supporting Hypotheses 2a and 2b. Our results were the same as the previous results on the transfer test. Both Richter et al. [11] and Xie et al. [12] found a small-to-medium cueing effect (*r* = 0.17 in the meta-analysis by Richter et al.; *g* = 0.36 in the meta-analysis by Xie et al.). Regarding the retention test, Xie et al.’s [12] meta-analysis and the current study both found that cues improved retention, but Xie et al. [12] found a medium-to-large cueing effect (*g* = 0.53) whereas our meta-analysis revealed a small-to-medium cueing effect. The reason for this discrepancy may be that we used different criteria than Xie et al. [12] did for including retrieved articles. Specifically, the articles included in the present study reported both SCL and learning outcomes, whereas Xie et al. [12] left SCL aside. Therefore the inclusion scope in our meta-analysis was different because studies with the measurement of retention scores but no SCL were excluded from our retention-related analysis. In addition, in support of Hypotheses 3a and 3b, *d*_SCL_ negatively predicted both *d*_retention_ (β = −0.70, *p* < 0.001) and *d*_transfer_ (β = −0.60, *p* < 0.001), suggesting that a lower cueing-related cognitive load meant higher scores on retention and transfer tests. Overall, results from the present study provided full support for CLT.

CLT is a plausible theory to explain the cueing effect. According to CLT, a reduction of subjective total cognitive load and an avoidance of cognitive overload would be expected in conditions with cues by virtue of their favorable external design, compared to conditions with no cues [5]. This assumption proved to be correct according to the results of our meta-analysis on the effects of cueing on SCL, retention and transfer. Thus, reducing cognitive load may be the crucial step in effective retention and comprehension in multimedia learning. However, to make a more direct inference about the cueing effect on learning outcomes based on the perspective of CLT, a further analysis of the relationship between cognitive load and scores of learning is probably requisite [23, 46]. Following CLT, the perceived total cognitive load would be negatively related to learning outcomes, such as retention and transfer performance [13]. Two meta-regression analyses in this study found this was indeed the case, again supporting CLT. These results were analogous to those of other empirical studies. Through two experiments, Huk et al. [23] discovered that students’ perceived cognitive load negatively correlated with both remembering and understanding. In Paik and Schraw’s study [46], learners were required to make a judgment of difficulty (JOD) after learning about a flushing toilet tank. Similarly, negative correlations were found between JOD and learners’ recall, as well as transfer performance.

Though the present study confirmed a reduction of total cognitive load (defined as subjective cognitive load in the form of mental effort, perceived difficulty, or their combination) in the cueing condition, it is seemingly impossible to draw conclusions about a specific type of cognitive load, i.e., ICL, ECL or GCL. Cueing is the consequence of external instructional design, but it may be related to both ECL and GCL [34]. For example, ECL would be decreased by reducing the unnecessary visual search when cues are provided in a given material (ICL is constant). However, it also could be that cueing increases GCL by optimizing the schema construction and automation.

It should be noted that the sensitivity of subjective ratings is critical for the measurement of cognitive load. SCL, especially mental effort, perceived difficulty, or their combination, is likely to be the most frequently used index of cognitive load [21, 38, 64, 65], but the sensitivity of measures of SCL has been questioned. After all, there are still quite a few empirical studies that have not found a reduction of SCL by cues as expected [4, 10, 36–41, 63]. There remain two possible explanations. First, there may be differences in participants’ understanding of the SCL questionnaire. Taking the question “How much effort did you have to invest to learn about the materials?” for example, some learners in the cueing condition might report that “I invested less effort because cues really worked” (as CLT would expect); others in the same condition could interpret the question in the opposite way, as “In an effort to comprehend the materials, I learned carefully” (not supporting CLT). Therefore, for the same question, learners in the cueing condition may respond differently from different viewpoints, probably decreasing the sensitivity of SCL.

Second, the effect of cueing on SCL may be moderated by external variables (e.g., dynamism of materials) that were not examined in the current analyses. For instance, cues have been found not to play a positive role in learning outcomes when the materials were dynamic [66]. Other studies have shown that when presenting materials in a static format, learners in the cueing condition outperformed those in the no-cueing condition [67]. A meta-analysis of cueing effects by Xie et al. [12] found that adding cues was beneficial for retention and transfer of knowledge when the multimedia material was static, whereas there were no significant effects on learning outcomes if the material was presented in a dynamic way. Taking CLT into consideration, these results could have been obtained because the elements in the dynamic materials overshadowed the effect of cueing on cognitive load, resulting in no learning improvement.

The current study presents the results of a meta-analysis adopting SCL as an index of cognitive load and scores on retention and transfer as indexes of learning outcomes based on CTL and, different from previous meta-analyses [11, 12], includes two meta-regression analyses focused on the relationship between SCL and learning outcomes, not only providing a more direct reference for CTL but also giving guidance on instructional design. Even so, several limitations should be acknowledged. First, we chose only SCL (specifically mental effort, perceived difficulty, or their combination) as the index of total cognitive load. Perhaps other measures would show additional unexpected but vital results. Second, we just focused on multimedia learning environments that contained both words and pictures. The effect of cueing when reading plain text (with no pictures) is also worthy of attention [12]. Third, though we argue that both mental effort and perceived difficulty measure the total cognitive load, it is not necessarily the case. After all, there are still many studies in which mental effort and perceived difficulty were used to measure ECL or GCL, leading to doubt about the measurement of cognitive load [19]. Moreover, differences between measurements of mental effort and measurements of perceived difficulty were ignored. It should be acknowledged that a learner’s rating of the perceived difficulty is not completely equivalent to the mental effort invested, though the two concepts are correlated. Van Gog and Paas [68] pointed out that mental effort was subordinate to a process, and contained more complex components than the task per se, whereas the perceived difficulty was mainly subject to the task. A potential example was that learners might not be motivated to invest sufficient mental effort when they perceived a specific task to be extremely difficult [68], leading to reversed scores of mental effort and the perceived difficulty scales. Thus, results concerning SCL in the present study must be treated with some caution.

All in all, in line with cognitive load theory, our meta-analysis and subsequent meta-regression analyses indicate that (a) cues can reduce subjective cognitive load, (b) cues can facilitate retention and transfer performance, and (c) the more SCL is reduced by cues, the better retention and transfer of multimedia learning. These results have clear theoretical and applied value.

## Supporting information

S1 Checklist PRISMA 2009 checklist. (DOC)

S1 Dataset (XLSX)
